# Coexisting Hearing Impairment in Children Presenting With Speech and Language Delay: A Case Series Highlighting the Importance of Routine Audiological Assessment

**DOI:** 10.1155/crpe/7681667

**Published:** 2026-09-29

**Authors:** Kyaw Linn, Win Lai May, Khin Nandar Kyaw, Aye Myat Sandar Win, Phyu Phyu Khaing Zin, Su Myat Soe, Hnin Wint Wint Aung

**Affiliations:** ^1^ Division of Paediatrics and Adolescent Medicine CLL HEALTH Yangon Myanmar; ^2^ CLL HEALTH Yangon Myanmar

**Keywords:** audiological assessment, case series, hearing impairment, neurodevelopmental disorder, speech delay

## Abstract

**Background:**

Speech and language delay is a common reason for referral to developmental pediatric services. Hearing impairment is an important and potentially manageable cause but may be overlooked because its clinical presentation often overlaps with autism spectrum disorder (ASD), attention‐deficit/hyperactivity disorder (ADHD), and other neurodevelopmental disorders. In Myanmar, where universal newborn hearing screening has not yet been routinely implemented, delayed recognition of hearing impairment remains an important clinical challenge.

**Case Presentation:**

We describe four children aged 5–7 years who were assessed at the Developmental Paediatrics Service of CLL HEALTH Community Paediatric Clinics, Yangon, Myanmar, after referral for speech delay, behavioral concerns, suspected ASD, attention difficulties, or school readiness assessment. Comprehensive developmental assessment together with formal audiological evaluation identified bilateral moderate sensorineural hearing loss in one child with language disorder, bilateral sensorineural hearing loss in a child with developmental coordination disorder and executive dysfunction, bilateral severe‐to‐profound hearing loss in a child initially suspected of having ADHD, and unilateral mild‐to‐moderate hearing loss in a child with ASD. None of the children had undergone newborn hearing screening. Hearing impairment either explained the presenting concerns or coexisted with neurodevelopmental disorders, contributing to communication, behavioral, and educational difficulties.

**Conclusion:**

This case series highlights the diagnostic challenges of recognizing hearing impairment in children presenting with developmental concerns in Myanmar, particularly in the absence of routine newborn hearing screening. Hearing impairment may mimic neurodevelopmental disorders, coexist with them, or contribute substantially to developmental difficulties. Routine audiological assessment should, therefore, be incorporated into the evaluation of all children presenting with speech and language delay or broader developmental concerns to facilitate timely diagnosis and appropriate multidisciplinary intervention.

## 1. Introduction

Speech and language delay is one of the most common reasons for referral to developmental pediatric services. The underlying causes are diverse and include hearing impairment, developmental language disorder, intellectual disability, autism spectrum disorder (ASD), attention‐deficit/hyperactivity disorder (ADHD), and other neurodevelopmental disorders [1]. Identifying the underlying cause is essential because management, prognosis, and long‐term developmental outcomes differ considerably according to the diagnosis.

Hearing impairment is an important and potentially manageable cause of speech and language delay. When identified early, interventions such as hearing amplification, speech and language therapy, and appropriate educational support can substantially improve communication and developmental outcomes. However, recognizing hearing impairment is not always straightforward. Children may present with delayed speech, poor response to their name, apparent inattention, behavioral difficulties, or impaired social interaction—features that overlap with those seen in ASD and other neurodevelopmental disorders. In addition, hearing impairment and neurodevelopmental disorders may coexist, further complicating the diagnostic process [2].

Universal newborn hearing screening has enabled early detection of congenital hearing loss in many high‐income countries [3]. In contrast, routine newborn hearing screening has not yet been widely implemented in many low‐ and middle‐income countries, including Myanmar. As a result, children with hearing impairment may first present to developmental services several years later with speech delay, behavioral concerns, learning difficulties, or suspected neurodevelopmental disorders rather than with concerns about hearing itself [2]. This delay may result in missed opportunities for intervention during the critical period of speech and language development.

For clinicians, the challenge is not simply to identify hearing impairment but also to determine whether it alone accounts for the child’s presentation or whether it coexists with an underlying neurodevelopmental disorder. Failure to recognize either condition may delay appropriate intervention and adversely affect communication, learning, and psychosocial development [2].

This case series describes four children referred to a developmental pediatric clinic in Myanmar for speech delay, behavioral concerns, suspected ASD, attention difficulties, or school readiness assessment. Comprehensive developmental assessment together with formal audiological evaluation identified hearing impairment as either the primary explanation for the presenting concerns or an important coexisting condition. Through these cases, we aim to highlight the continued problem of delayed recognition of childhood hearing impairment in settings without universal newborn hearing screening, demonstrate that hearing impairment remains an important and potentially manageable cause of speech and language delay, and illustrate the diagnostic challenges of distinguishing hearing impairment from, or recognizing its coexistence with, neurodevelopmental disorders. We hope these cases reinforce the need for routine audiological assessment in every child presenting with speech and language delay or broader developmental concerns, regardless of whether a neurodevelopmental disorder is suspected.

## 2. Case Series

The following four cases were identified from routine clinical practice at the Developmental Paediatrics Service of CLL HEALTH Community Paediatric Clinics, Yangon, Myanmar. They were selected as a convenience sample because they represent different clinical presentations of hearing impairment encountered among children referred for developmental assessment and illustrate common diagnostic challenges in routine practice. All children underwent comprehensive developmental assessment together with formal audiological evaluation. Table 1 summarizes the audiological assessment methods and findings for Cases 1, 2, and 4, while Table 2 summarizes the demographic characteristics, referral concerns, final diagnoses, audiological diagnoses, and clinical significance for all four cases.

**TABLE 1:** Audiological assessment methods and findings for Cases 1, 2, and 4.

Audiological assessment methods	Case 1	Case 2	Case 4
Immittance audiometry	Bilateral Type A shallow tympanograms, consistent with reduced middle‐ear compliance.	Bilateral Type A shallow tympanograms consistent with normal middle ear pressure and low compliance.	Type C tympanogram consistent with negative middle ear pressure and low compliance in Rt ear.
			Type A tympanogram consistent with normal middle ear pressure and compliance in Lt ear.

Otoacoustic emissions (OAE) (TEOAE + DPOAE)	Absent bilaterally	Absent bilaterally	Lt ear consistent with normal or near normal cochlear function.
			Absent in the right ear, which may be attributable to middle‐ear dysfunction associated with the Type C tympanogram.

Auditory brain stem response (ABR)	“Refer” result in both ears	“Refer” for both ears	Pass for her Lt ear. Refer for her Rt ear.

Auditory steady state response (ASSR)	In Rt ear, responses were obtained at 60 dB nHL at 1 kHz and 50 dB nHL at 2 and 4 kHz.	Rt ear showed 65 dB nHL response at 4 KHz, 70 dB nHL response at 1 KHz, 2 KHz and also at 500 Hz.	Right ear showed mild to moderate hearing loss.
	In Lt ear, responses were obtained at 60 dB nHL at 500 Hz and 50 dB nHL at 1, 2, and 4 kHz.	Lt ear showed 40 dB nHL response at 2 KHz and 4 KHz, 65 dB nHL response at 500 Hz and 1 KHz	Lt ear showed essentially normal hearing.

Pure tone audiometry		Rt ear‐25 dB	
		Lf ear‐23 dB	

*Note:* Pure‐tone audiometry was obtained only in Case 2 because reliable behavioral pure‐tone thresholds could not be obtained in Cases 1 and 4; objective electrophysiological measures were, therefore, used to estimate hearing thresholds. Case 3 was not included in Table 1 because she had an established diagnosis of bilateral severe‐to‐profound sensorineural hearing loss prior to referral to our developmental service and was already receiving hearing‐aid‐based intervention.

**TABLE 2:** Summary of clinical characteristics.

Case	Age/sex	Primary referral concern	Final clinical diagnosis	Audiological diagnosis	Clinical significance
1	5‐year‐old male	Speech delay, possible ASD	Language disorder secondary to hearing impairment with pragmatic language difficulties	Bilateral moderate sensorineural hearing loss	Hearing loss contributed to speech delay and initially raised concern for ASD.
2	7‐year–7‐month‐old male	Global developmental delay, attention difficulties, school readiness	Developmental coordination disorder, developmental language disorder, and executive dysfunction	Bilateral sensorineural hearing loss (right worse than left) on ASSR/ABR; considered mild on subsequent pure tone audiometry (ENT review)	Hearing impairment coexisted with neurodevelopmental disorders and compounded communication and learning difficulties.
3	6‐year–6‐month‐old female	Suspected ADHD because of poor attention during therapy	Speech and language delay secondary to bilateral severe‐to‐profound SNHL	Bilateral severe‐to‐profound sensorineural hearing loss	Apparent attention difficulties were attributable to hearing impairment rather than ADHD.
4	5‐year–10‐month‐old female	Hyperactivity, suspected ASD, school readiness	ASD (Level 1 support)	Unilateral mild‐to‐moderate hearing loss (right ear)	Hearing impairment coexisted with ASD and contributed to communication and school readiness difficulties.

### 2.1. Case 1

#### 2.1.1. Presentation

A 5‐year‐old boy was referred because of significant expressive language delay, limited reciprocal verbal interaction, and an inconsistent response to his name. Developmental concerns became apparent at 3 years of age following several hospital admissions for asthma and surgery at 2 years of age, after which he developed marked social anxiety. He was raised in a multilingual household (Myanmar and Kayin). Expressive language was limited to approximately three functional words and pointing although receptive language was relatively preserved, with the ability to follow three‐step unrelated commands. He demonstrated good rote memory for numbers and the English alphabet but had difficulty learning nursery rhymes and the Myanmar alphabet.

#### 2.1.2. Clinical Findings

He was underweight (< third percentile) and short for age (fifth percentile), with no dysmorphic features. During the clinic assessment, social reciprocity and eye contact appeared reduced. However, home videos showed frequent joint attention, social referencing, and imaginative play, making ASD less likely. Drooling during effortful speech suggested an associated oral‐motor coordination difficulty. A formal speech production assessment was not performed at the initial evaluation; formal speech‐language assessment was recommended as part of subsequent multidisciplinary management.

#### 2.1.3. Investigations

Audiological assessment demonstrated bilateral moderate sensorineural hearing loss. Blood investigations showed mild hypochromic microcytic anemia (hemoglobin 10.8 g/dL), while thyroid function and ferritin levels were normal.

#### 2.1.4. Clinical Impression

The final diagnosis was language disorder secondary to bilateral moderate sensorineural hearing loss, presenting with pragmatic language difficulties together with an oral‐motor component.

#### 2.1.5. Follow‐Up

At follow‐up, he continued to demonstrate intact joint attention, positive social referencing, purposeful imaginative play, and the ability to follow three‐step directions. Management included further audiological review, initiation of hearing aids and Auditory–Verbal Therapy, classroom visual and acoustic accommodations, and dietary iron supplementation. Comprehensive developmental re‐evaluation was planned after 3 months.

### 2.2. Case 2

#### 2.2.1. Presentation

A 7‐year‐7‐month‐old boy with previous diagnosis of global developmental delay, mild ASD, and ADHD was referred for diagnostic clarification and assessment of school readiness. Previous treatment with methylphenidate and risperidone had been discontinued because of adverse effects. His first meaningful words emerged at approximately 4 years of age, but he continued to have marked pragmatic language difficulties, including inappropriate pronoun use and limited spontaneous conversation.

#### 2.2.2. Clinical Findings

Neurological examination demonstrated mild distal upper‐limb hypotonia and weakness without evidence of a generalized neuromuscular disorder. Standardized motor assessment revealed severe deficits in manual dexterity, balance, and visual‐motor integration. Overall developmental functioning showed an uneven profile, with relative strengths in rote auditory memory and word decoding but marked executive dysfunction.

#### 2.2.3. Investigations

Formal audiological assessment confirmed bilateral sensorineural hearing loss, which was more pronounced in the right ear. Laboratory investigations identified mild vitamin D deficiency, hypocalcaemia, and hypomagnesaemia.

#### 2.2.4. Clinical Impression

The child was diagnosed with developmental coordination disorder, developmental language disorder, executive dysfunction with ADHD traits, and bilateral sensorineural hearing loss.

#### 2.2.5. Follow‐Up

Hearing aids were not prescribed following ENT assessment, as pure tone audiometry demonstrated only mild hearing loss. Given the discordance between electrophysiological and behavioral hearing assessments, repeat audiological evaluation was recommended to clarify his functional hearing status and guide further management. He demonstrated improvement in gross motor balance, fine motor praxis, self‐care skills, emotional regulation, and reduction of passive screen time. Persistent difficulties included macrographia, slow sentence‐level writing, expressive speech articulation difficulties, and situational noncompliance and hyperactivity, particularly during occupational therapy and academic tutoring.

### 2.3. Case 3

#### 2.3.1. Presentation

A 6‐year‐6‐month‐old girl with known bilateral severe‐to‐profound sensorineural hearing loss was referred because of concerns regarding a short attention span during Auditory–Verbal Therapy. She had been fitted with bilateral hearing aids at 4 years of age and communicated using three‐ to four‐word sentences. Her past medical history was significant only for neonatal jaundice requiring phototherapy.

#### 2.3.2. Clinical Findings

She was cooperative throughout the assessment, maintained good eye contact, and sustained attention for approximately 30 min during structured activities. Although she experienced difficulties participating in group activities at preschool, these difficulties appeared to be related to her hearing impairment rather than to deficits in attention or social communication.

#### 2.3.3. Investigations

Assessment using the Vanderbilt ADHD Rating Scale did not support a diagnosis of ADHD.

#### 2.3.4. Clinical Impression

The final diagnosis was speech and language delay secondary to bilateral severe‐to‐profound sensorineural hearing loss, with no evidence of coexisting ADHD.

#### 2.3.5. Follow‐Up

At follow‐up, she demonstrated emerging speech‐motor control for early‐developing sounds (/ *p*/,/*t*/,/*s*/,/*m*/,/*n*/) and responded well to tactile cueing. However, auditory‐only word recognition remained below 50%, with minimal additional benefit from speech reading. Given her limited auditory access despite hearing‐aid use, cochlear implant evaluation was recommended, together with continued Auditory–Verbal Therapy and consistent daily hearing‐aid use pending further assessment.

### 2.4. Case 4

#### 2.4.1. Presentation

A 5‐year‐10‐month‐old girl was referred because of hyperactivity, inattention, learning inflexibility, and concerns regarding school readiness. Her developmental history included regression at approximately 2 years of age, characterized by loss of previously acquired two‐word phrases and reduced eye contact. She subsequently developed persistent climbing and jumping together with a preference for solitary play.

#### 2.4.2. Clinical Findings

She fulfilled DSM‐5 criteria for ASD requiring Level 1 support, with reduced social–emotional reciprocity, inconsistent joint attention, and restricted interests. Expressive language consisted mainly of three‐word phrases with immature pronoun use. Her body mass index was at the third percentile.

#### 2.4.3. Investigations

Audiological assessment showed a Type C tympanogram with absent otoacoustic emissions in the right ear. Auditory steady‐state response and auditory brainstem response testing confirmed mild‐to‐moderate hearing loss in the right ear, while hearing in the left ear was essentially normal.

#### 2.4.4. Clinical Impression

The final diagnosis was ASD requiring Level 1 support with coexisting hyperactivity and unilateral mild‐to‐moderate hearing loss, both contributing to communication difficulties and reduced school readiness.

#### 2.4.5. Follow‐Up

Following 2 weeks of medical therapy, she underwent endoscopic otolaryngological assessment, which identified a bulge adjacent to the tympanic membrane associated with reduced hearing, together with nasal congestion. Nasal decongestant therapy was prescribed for 1 month. At ENT follow‐up 2 weeks later, otoscopy showed near‐complete resolution of the right‐sided bulge and a clear left ear although nasal obstruction persisted. She continued behavioral auditory‐response training at school, with reported improvement in responsiveness during hearing assessment and overall behavior. A repeat formal audiological assessment following resolution of the middle‐ear abnormality had not yet been completed at the time of manuscript preparation and was planned as part of follow‐up.

## 3. Discussion

This case series illustrates the varied ways in which hearing impairment may present in children referred for developmental assessment. In one child, hearing loss contributed to concerns regarding ASD; in another, attention difficulties were better explained by hearing impairment than ADHD; and in two children, hearing impairment coexisted with neurodevelopmental disorders. Together, these cases show that hearing impairment may mimic, coexist with, or compound neurodevelopmental disorders, making diagnosis challenging in routine clinical practice.

The overlap between hearing impairment and neurodevelopmental disorders is well recognized. Delayed speech, reduced response to name, impaired social communication, apparent inattention, behavioral difficulties, and learning problems are common features of both conditions [2, 4–7]. As a result, hearing impairment may be overlooked when behavioral features are attributed to ASD, ADHD, or other developmental disorders. This phenomenon, often described as diagnostic overshadowing, has been reported previously in children whose hearing impairment was initially mistaken for autism [2, 4]. Case 1 illustrates this challenge well. Although reduced eye contact and limited social reciprocity initially raised concerns about ASD, naturalistic observations showed preserved joint attention and imaginative play, while audiological assessment identified bilateral sensorineural hearing loss as an important contributor to the child’s communication difficulties.

Equally important, hearing impairment and neurodevelopmental disorders should not be considered mutually exclusive. Cases 2 and 4 demonstrate that hearing loss may coexist with developmental coordination disorder, developmental language disorder, executive dysfunction, or ASD. In these children, hearing impairment did not fully account for the developmental profile but added to existing communication and learning difficulties. Previous studies have also shown that hearing impairment is more common among children with autism and other developmental disabilities than in the general pediatric population [5–7]. Recognizing both conditions is important because management must address each of them rather than assuming that a single diagnosis explains all developmental concerns.

Our experience also supports performing developmental and audiological assessments in parallel. Waiting until one assessment is completed before arranging the other may unnecessarily delay intervention. In children presenting with speech delay or developmental concerns, information from developmental history, clinical observation, audiological assessment, educational reports, and standardized developmental testing should be considered together [8–11]. This approach helps define the contribution of hearing impairment to the child’s presentation and guides appropriate intervention.

An interesting finding in this series was that parents rarely suspected hearing impairment. Instead, they sought medical attention because of delayed speech, behavioral concerns, poor attention, or difficulties at school. Similar findings have been reported previously, with many parents of children with speech delay expressing no concern about hearing despite objective evidence of hearing impairment [12]. This highlights the importance of routine audiological assessment in all children presenting with speech and language delay, regardless of parental concern or the presence of another developmental diagnosis.

None of the children in this series had undergone newborn hearing screening, and hearing impairment was recognized only between five and seven years of age. In countries where universal newborn hearing screening has been established, congenital hearing loss is usually identified much earlier [3]. In Myanmar and many other resource‐limited settings, however, developmental clinics may represent the first opportunity for comprehensive hearing assessment. Although these children were diagnosed later than recommended, appropriate hearing rehabilitation, speech and language therapy, educational support, and multidisciplinary intervention still offered meaningful opportunities to improve communication and participation.

Awareness of the relationship between hearing impairment and speech and language development among frontline healthcare professionals is also important. In settings without universal newborn hearing screening, general practitioners, general pediatricians, primary‐care clinicians, preschool teachers, and other professionals may be the first to encounter children with delayed communication. Education of these professionals regarding hearing‐related presentations and clear referral pathways for audiological assessment may, therefore, facilitate earlier recognition. Although professional awareness was not formally evaluated in this case series, strengthening awareness and referral pathways represents an important area for service development.

## 4. Practical Recommendations for Resource‐Limited Settings

In settings where universal newborn hearing screening is not routinely available and specialist audiology resources are limited, hearing assessment should be incorporated into the clinical pathway for children presenting with speech or language delay and broader developmental concerns. At the first point of contact, clinicians should obtain a focused hearing history, including response to name and environmental sounds, previous otitis media, risk factors for hearing impairment, and parental concerns, together with otoscopic examination where feasible. Children with speech or language delay should be referred for formal audiological assessment irrespective of whether another neurodevelopmental disorder is suspected.

Where audiology capacity is limited, referral pathways may prioritize children with significant language delay, inconsistent auditory responsiveness, developmental regression, recurrent middle‐ear disease, recognized risk factors for hearing loss, or concerns raised by caregivers or educational professionals. Age and developmentally appropriate behavioral audiometry should be used where reliable responses can be obtained, supplemented by tympanometry and otoacoustic emissions. Objective electrophysiological assessment, such as ABR or ASSR, may be required when behavioral testing is unreliable or inconclusive. Developmental and audiological assessments should, where possible, proceed in parallel rather than sequentially to minimize delays in intervention. Clear referral pathways between primary care, developmental pediatrics, ENT, audiology, speech and language services, and educational providers may further facilitate timely diagnosis and multidisciplinary management.

This study has several limitations. It is a small case series from a single community developmental pediatric service, and long‐term developmental outcomes were not available. The cases were not intended to estimate the prevalence of hearing impairment among children with developmental concerns. Instead, they illustrate common diagnostic challenges encountered in everyday clinical practice and reinforce the importance of considering hearing impairment throughout the developmental assessment process.

## 5. Conclusion

This case series demonstrates that hearing impairment remains an important consideration in children presenting with speech and language delay or developmental concerns. Hearing impairment may be the primary cause of language delay, mimic neurodevelopmental disorders because of overlapping behavioral features, or coexist with conditions such as ASD and developmental coordination disorder. These possibilities should be considered routinely during assessment.

In settings where universal newborn hearing screening is not yet routinely available, delayed diagnosis remains a significant challenge. Our cases suggest that formal audiological assessment should be incorporated into the routine evaluation of all children with speech delay, behavioral concerns, or suspected neurodevelopmental disorders, regardless of parental concern or the apparent presence of another diagnosis. Early recognition, combined with comprehensive developmental assessment and multidisciplinary intervention, offers the best opportunity to improve communication, educational participation, and long‐term developmental outcomes.

## Author Contributions

Win Lai May and Khin Nandar Kyaw contributed to the conception of the case series, collected and interpreted the clinical data, and drafted the initial manuscript. Kyaw Linn critically reviewed, revised, and edited the manuscript for important intellectual content. Aye Myat Sandar Win, Phyu Phyu Khaing Zin, Su Myat Soe, and Hnin Wint Wint Aung ensured clinical data curation.

## Funding

No funding was received for this manuscript.

## Disclosure

All authors have reviewed and approved the final version of the manuscript and agreed to be accountable for all aspects of the work.

## Ethics Statement

Formal ethics committee approval was not required for this case series in accordance with the institutional policy of CLL HEALTH, as it comprised a retrospective description of cases identified during routine clinical care and involved no research‐related intervention or alteration of patient management. Written informed consent for inclusion in the case series and publication of the relevant anonymized clinical information was obtained from the parents or legal guardians of all children. Patient confidentiality was maintained, and all identifying information was removed. The case series was conducted and reported in accordance with applicable institutional requirements and the ethical principles of the Declaration of Helsinki.

## Conflicts of Interest

The authors declare no conflicts of interest.

## Data Availability

The data that support the findings of this study are available from the corresponding author upon reasonable request.
